# Bis(μ-3-hy­droxy­benzoato)-κ^3^ *O* ^1^,*O* ^1′^:*O* ^1^;κ^3^ *O* ^1^:*O* ^1^,*O* ^1′^-bis­[(3-hy­droxy­benzoato-κ^2^ *O*,*O*′)(iso­nicotinamide-κ*N* ^1^)cadmium] tetra­hydrate

**DOI:** 10.1107/S160053681200219X

**Published:** 2012-01-21

**Authors:** Ibrahim Göker Zaman, Nagihan Çaylak Delibaş, Hacali Necefoğlu, Tuncer Hökelek

**Affiliations:** a Department of Chemistry, Kafkas University, 36100 Kars, Turkey; b Department of Physics, Sakarya University, 54187 Esentepe, Sakarya, Turkey; c Department of Physics, Hacettepe University, 06800 Beytepe, Ankara, Turkey

## Abstract

In the title centrosymmetric binuclear Cd^II^ compound, [Cd_2_(C_7_H_5_O_3_)_4_(C_6_H_6_N_2_O)_2_]·4H_2_O, the six-coordinated Cd^II^ atom is chelated by the carboxyl­ate groups of the two 3-hy­droxy­benzoate (HB) anions; the two monomeric units are bridged through the two O atoms of the two carboxyl­ate groups. In the crystal, O—H⋯O, N—H⋯O and C—H⋯O hydrogen bonds link the mol­ecules into a three-dimensional network. π–π Contacts between the pyridine rings and between the benzene rings [centroid-centroid distances = 3.770 (1), 3.769 (1) and 3.632 (1) Å] may further stabilize the structure.

## Related literature

For coordination complexes of niacin, see: Krishnamachari (1974 ▶) and for coordination complexes of *N*,*N*-diethyl­nicotinamide, see: Bigoli *et al.* (1972 ▶). For related structures, see: Greenaway *et al.* (1984 ▶); Hökelek & Necefoğlu (1996 ▶); Hökelek *et al.* (2009*a* ▶,*b* ▶,*c* ▶,*d* ▶, 2010*a* ▶,*b* ▶).
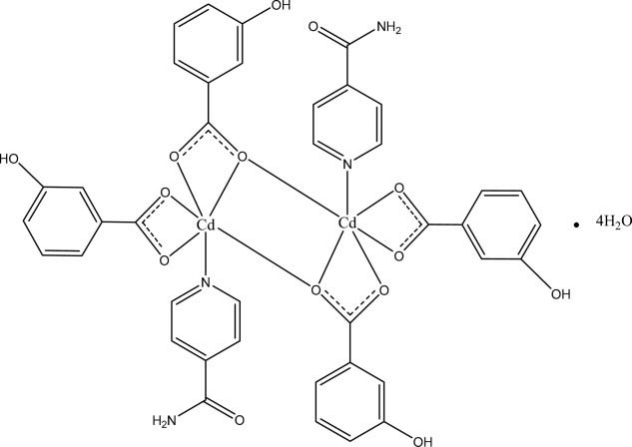


## Experimental

### 

#### Crystal data

[Cd_2_(C_7_H_5_O_3_)_4_(C_6_H_6_N_2_O)_2_]·4H_2_O
*M* *_r_* = 1333.82
Triclinic, 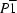

*a* = 9.1131 (3) Å
*b* = 11.5757 (4) Å
*c* = 13.6810 (4) Å
α = 94.032 (2)°
β = 97.762 (2)°
γ = 109.190 (3)°
*V* = 1340.35 (8) Å^3^
*Z* = 1
Mo *K*α radiation
μ = 0.88 mm^−1^
*T* = 100 K
0.37 × 0.29 × 0.19 mm

#### Data collection

Bruker Kappa APEXII CCD area-detector diffractometer
Absorption correction: multi-scan (*SADABS*; Bruker, 2005 ▶) *T*_min_ = 0.744, *T*_max_ = 0.846
20433 measured reflections
4842 independent reflections
4689 reflections with *I* > 2σ(*I*)
*R*_int_ = 0.024

#### Refinement

*R*[*F* ^2^ > 2σ(*F* ^2^)] = 0.017
*wR*(*F* ^2^) = 0.043
*S* = 1.07
4842 reflections
385 parameters
4 restraints
H atoms treated by a mixture of independent and constrained refinement
Δρ_max_ = 0.38 e Å^−3^
Δρ_min_ = −0.38 e Å^−3^

### 

Data collection: *APEX2* (Bruker, 2007 ▶); cell refinement: *SAINT* (Bruker, 2007 ▶); data reduction: *SAINT*; program(s) used to solve structure: *SHELXS97* (Sheldrick, 2008 ▶); program(s) used to refine structure: *SHELXL97* (Sheldrick, 2008 ▶); molecular graphics: *ORTEP-3 for Windows* (Farrugia, 1997 ▶); software used to prepare material for publication: *WinGX* (Farrugia, 1999 ▶) and *PLATON* (Spek, 2009 ▶).

## Supplementary Material

Crystal structure: contains datablock(s) I, global. DOI: 10.1107/S160053681200219X/su2364sup1.cif

Structure factors: contains datablock(s) I. DOI: 10.1107/S160053681200219X/su2364Isup2.hkl

Additional supplementary materials:  crystallographic information; 3D view; checkCIF report

## Figures and Tables

**Table 1:** Hydrogen-bond geometry (Å, °)

*D*—H⋯*A*	*D*—H	H⋯*A*	*D*⋯*A*	*D*—H⋯*A*
N2—H2*A*⋯O8^i^	0.86	2.12	2.9158 (19)	153
N2—H2*B*⋯O2^ii^	0.86	2.00	2.8290 (18)	160
O3—H3*O*⋯O8^iii^	0.82	1.91	2.7248 (16)	173
N4—H4*A*⋯O2^iv^	0.86	2.20	3.0404 (18)	164
N4—H4*B*⋯O9^v^	0.86	2.01	2.846 (2)	166
O6—H6*O*⋯O10^v^	0.82	1.91	2.7043 (18)	163
O9—H91⋯O10^vi^	0.79 (2)	2.03 (2)	2.819 (2)	173 (2)
O9—H92⋯O7^vii^	0.78 (2)	2.29 (2)	2.9508 (19)	143 (2)
O10—H101⋯O3^vi^	0.81 (2)	2.17 (2)	2.8976 (19)	150 (2)
O10—H102⋯O7^viii^	0.81 (2)	1.89 (2)	2.6876 (18)	165 (3)
C14—H14⋯O1^ix^	0.93	2.28	3.197 (2)	171
C19—H19⋯O1	0.93	2.54	3.161 (2)	124
C21—H21⋯O6^x^	0.93	2.49	3.128 (2)	126
C22—H22⋯O6^x^	0.93	2.58	3.163 (2)	121
C24—H24⋯O9^v^	0.93	2.32	3.222 (2)	164

Symmetry codes: (i) 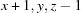; (ii) 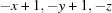; (iii) 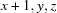; (iv) 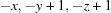; (v) 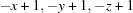; (vi) 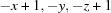; (vii) 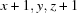; (viii) 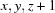; (ix) 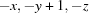; (x) 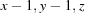.

**Fig. 1.**
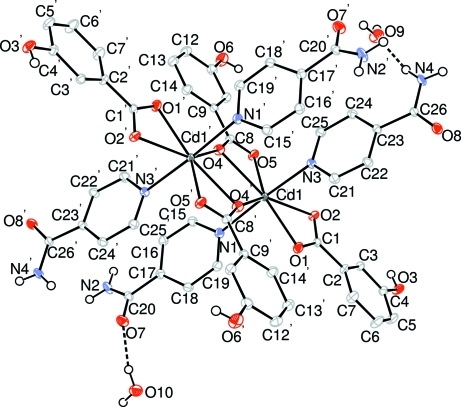
The molecular structure of the title compound, with the atom-numbering and displacement ellipsoids drawn at the 50% probability level [Primed atoms are generated by the symmetry code: (') -x, -y+1, -z; Hydrogen bonds are shown as dashed lines; C-bound H atoms have been omitted for clarity].

## supplementary crystallographic information

### Comment

As a part of our ongoing investigation on transition metal complexes of nicotinamide (NA), one form of niacin (Krishnamachari, 1974), and/or the nicotinic acid derivative *N*,*N*-diethylnicotinamide (DENA), an important respiratory stimulant (Bigoli *et al.*, 1972), the title compound was synthesized and its crystal structure is reported herein.

The title compound consists of dimeric units located about an inversion centre and is made up of two Cd^2+^ cations, four 3-hydroxybenzoate (HB) anions, two isonicotinamide (INA) ligands and four uncoordinated water molecules (Fig. 1). Each Cd^II^ atom is chelated by the carboxylate O atoms of the two HB anions, and the two monomeric units are bridged through the two O atoms of the two carboxylate groups about the inversion center. The coordination number of each Cd^II^ atom is six. The Cd1···Cd1^i^ distance is 3.818 (1) Å and O4-Cd1-O4^i^ angle is 75.96 (4)° [symmetry code: (i) -x, -y+1, -z].

The Cd-O bond lengths vary from 2.3039 (11) to 2.5562 (11) Å with an average Cd-O value of 2.4033 (11) Å. The Cd atom is displaced out of the mean planes of the carboxylate groups, (O1/C1/O2) and (O4/C8/O5), by -0.0053 (1) and 0.0965 (1) Å, respectively. The O1-Cd1-O2 and O4-Cd1-O5 bond angles are 53.71 (4) and 54.59 (4) °, respectively. The corresponding O-M-O (where M is a metal) angles are 55.71 (5)° and 117.52 (4)° in [Cd_2_(MAB)_4_(NA)_2_(H_2_O)_2_] (Hökelek *et al.*, 2010*a*), 55.96 (4)° and 53.78 (4)° in [Cd_2_(DMAB)_4_(NA)_2_(H_2_O)_2_] (Hökelek *et al.*, 2010*b*), 52.91 (4)° and 53.96 (4)° in [Cd(FB)_2_(INA)_2_(H_2_O)].H_2_O (Hökelek *et al.*, 2009*a*), 60.70 (4)° in [Co(DMAB)_2_(INA)(H_2_O)_2_] (Hökelek *et al.*, 2009*b*), 58.45 (9)° in [Mn(DMAB)_2_(INA)(H_2_O)_2_] (Hökelek *et al.*, 2009*c*), 60.03 (6)° in [Zn(MAB)_2_(INA)_2_].H_2_O (Hökelek *et al.*, 2009*d*), 58.3 (3)° in [Zn_2_(DENA)_2_(HB)_4_].2H_2_O (Hökelek & Necefoğlu, 1996) [where NA, INA, DENA, HB, FB, MAB and DMAB are nicotinamide, isonicotinamide, *N*,*N*-diethylnicotinamide, 4-hydroxybenzoate, 4-formylbenzoate, 4-methylaminobenzoate and 4-dimethylaminobenzoate, respectively] and 55.2 (1)° in [Cu(Asp)_2_(py)_2_] (where Asp is acetylsalicylate and py is pyridine) (Greenaway *et al.*, 1984).

The dihedral angles between the planar carboxylate groups and the adjacent benzene rings A (C2-C7) and B (C9-C14) are 10.25 (10) and 0.86 (11) °, respectively, while those between rings A, B, C (N1/C15-C19), D (N3/C21-C25), E (Cd1/O1/O2/C1) and F (Cd1/O4/O5/C8) are A/B = 3.13 (4), A/C = 73.27 (5), A/D = 77.13 (4), B/C = 70.25 (5), B/D = 74.27 (4), C/D = 9.07 (5) and E/F = 9.99 (4) °.

In the crystal, intermolecular O-H···O, N-H···O and C-H···O hydrogen bonds (Table 1) link the molecules into a three-dimensional network. The π–π contacts between the pyridine rings and between the benzene rings, Cg3—Cg4^i^, Cg1—Cg2^ii^ and Cg2—Cg2^iii^ [symmetry codes: (i) -x, -y+1, -z, (ii) x, +y-1, z, (iii) -x+1, -y+2, -z, where Cg1, Cg2, Cg3 and Cg4 are the centroids of the rings A (C2-C7), B (C9-C14), C (N1/C15-C19) and D (N3/C21-C25), respectively] further stabilize the crystal structure, with centroid-centroid distances of 3.770 (1), 3.769 (1) and 3.632 (1) Å, respectively.

### Experimental

The title compound was prepared by the reaction of 3CdSO_4_.8H_2_O (0.428 g, 5 mmol) in H_2_O (100 ml) and INA (1.220 g, 10 mmol) in H_2_O (50 ml) with sodium 3-hydroxybenzoate (1.601 g, 10 mmol) in H_2_O (100 ml). The mixture was filtered and set aside to crystallize at ambient temperature for five weeks, giving colourless block-like crystals.

### Refinement

Atoms H91, H92, H101 and H102 (for H_2_O) were located in a difference Fourier map and were freely refined. The remaining H-atoms were included in calculated postions and constrained to ride on their parent atoms: O—H = 0.82 Å, N—H = 0.86 Å, C—H = 0.93 Å, with *U*_iso_(H) = k × *U*_eq_(C,O,N), where k = 1.5 for OH H-atoms and k = 1.2 for all other H-atoms.

### Crystal data

**Table:**

[Cd_2_(C_7_H_5_O_3_)_4_(C_6_H_6_N_2_O)_2_]·4H_2_O	*Z* = 1
*M**_r_* = 1333.82	*F*(000) = 676
Triclinic, *P*1	*D*_x_ = 1.652 Mg m^−^^3^
Hall symbol: -P 1	Mo *K*α radiation, λ = 0.71073 Å
*a* = 9.1131 (3) Å	Cell parameters from 9327 reflections
*b* = 11.5757 (4) Å	θ = 2.4–28.5°
*c* = 13.6810 (4) Å	µ = 0.88 mm^−^^1^
α = 94.032 (2)°	*T* = 100 K
β = 97.762 (2)°	Block, colorless
γ = 109.190 (3)°	0.37 × 0.29 × 0.19 mm
*V* = 1340.35 (8) Å^3^	

### Data collection

**Table:**

Bruker Kappa APEXII CCD area-detector diffractometer	4842 independent reflections
Radiation source: fine-focus sealed tube	4689 reflections with *I* > 2σ(*I*)
graphite	*R*_int_ = 0.024
φ and ω scans	θ_max_ = 25.3°, θ_min_ = 2.4°
Absorption correction: multi-scan (*SADABS*; Bruker, 2005)	*h* = −10→10
*T*_min_ = 0.744, *T*_max_ = 0.846	*k* = −13→13
20433 measured reflections	*l* = −16→16

### Refinement

**Table:**

Refinement on *F*^2^	Secondary atom site location: difference Fourier map
Least-squares matrix: full	Hydrogen site location: inferred from neighbouring sites
*R*[*F*^2^ > 2σ(*F*^2^)] = 0.017	H atoms treated by a mixture of independent and constrained refinement
*wR*(*F*^2^) = 0.043	*w* = 1/[σ^2^(*F*_o_^2^) + (0.0176*P*)^2^ + 0.8878*P*] where *P* = (*F*_o_^2^ + 2*F*_c_^2^)/3
*S* = 1.07	(Δ/σ)_max_ = 0.001
4842 reflections	Δρ_max_ = 0.38 e Å^−^^3^
385 parameters	Δρ_min_ = −0.38 e Å^−^^3^
4 restraints	Extinction correction: *SHELXL97* (Sheldrick, 2008), Fc^*^=kFc[1+0.001xFc^2^λ^3^/sin(2θ)]^-1/4^
Primary atom site location: structure-invariant direct methods	Extinction coefficient: 0.0220 (6)

### Special details

**Table:**

Geometry. All esds (except the esd in the dihedral angle between two l.s. planes) are estimated using the full covariance matrix. The cell esds are taken into account individually in the estimation of esds in distances, angles and torsion angles; correlations between esds in cell parameters are only used when they are defined by crystal symmetry. An approximate (isotropic) treatment of cell esds is used for estimating esds involving l.s. planes.
Refinement. Refinement of F^2^ against ALL reflections. The weighted R-factor wR and goodness of fit S are based on F^2^, conventional R-factors R are based on F, with F set to zero for negative F^2^. The threshold expression of F^2^ > 2sigma(F^2^) is used only for calculating R-factors(gt) etc. and is not relevant to the choice of reflections for refinement. R-factors based on F^2^ are statistically about twice as large as those based on F, and R- factors based on ALL data will be even larger.

### Fractional atomic coordinates and isotropic or equivalent isotropic displacement parameters (Å^2^)

**Table:**

	*x*	*y*	*z*	*U*_iso_*/*U*_eq_	
Cd1	0.119418 (12)	0.446033 (9)	0.100945 (8)	0.00971 (5)	
O1	0.09141 (13)	0.24933 (10)	0.13704 (8)	0.0150 (2)	
O2	0.27476 (13)	0.38791 (10)	0.24766 (8)	0.0146 (2)	
O3	0.51014 (14)	0.13170 (11)	0.46289 (9)	0.0180 (2)	
H3O	0.5619	0.2055	0.4714	0.027*	
O4	0.11035 (13)	0.62981 (10)	0.02257 (8)	0.0154 (2)	
O5	0.29795 (13)	0.64381 (10)	0.14674 (8)	0.0154 (2)	
O6	0.62271 (15)	1.09932 (11)	0.21834 (9)	0.0241 (3)	
H6O	0.6453	1.0594	0.2615	0.036*	
O7	0.27397 (14)	0.21759 (11)	−0.35970 (9)	0.0195 (3)	
O8	−0.33406 (13)	0.37934 (10)	0.50232 (8)	0.0165 (2)	
O9	1.00445 (16)	0.17721 (12)	0.47999 (10)	0.0247 (3)	
H91	0.925 (2)	0.1261 (18)	0.4526 (16)	0.034*	
H92	1.057 (2)	0.153 (2)	0.5173 (16)	0.034*	
O10	0.29077 (15)	−0.00976 (12)	0.61445 (10)	0.0227 (3)	
H101	0.353 (2)	−0.021 (2)	0.5812 (16)	0.034*	
H102	0.298 (3)	0.0623 (16)	0.6162 (17)	0.034*	
N1	0.24228 (15)	0.40441 (12)	−0.02882 (10)	0.0137 (3)	
N2	0.48486 (16)	0.39211 (13)	−0.33960 (10)	0.0192 (3)	
H2A	0.5065	0.3769	−0.3974	0.023*	
H2B	0.5429	0.4581	−0.3015	0.023*	
N3	−0.02547 (15)	0.46803 (12)	0.22393 (10)	0.0124 (3)	
N4	−0.19976 (16)	0.58150 (13)	0.54420 (10)	0.0156 (3)	
H4A	−0.2399	0.5830	0.5975	0.019*	
H4B	−0.1338	0.6478	0.5297	0.019*	
C1	0.19456 (18)	0.27725 (14)	0.21455 (11)	0.0119 (3)	
C2	0.22146 (18)	0.17564 (14)	0.26777 (11)	0.0130 (3)	
C3	0.35124 (18)	0.20337 (14)	0.34290 (11)	0.0130 (3)	
H3	0.4177	0.2846	0.3613	0.016*	
C4	0.38114 (19)	0.10955 (15)	0.39013 (12)	0.0147 (3)	
C5	0.2811 (2)	−0.01149 (16)	0.36388 (13)	0.0205 (4)	
H5	0.3013	−0.0744	0.3957	0.025*	
C6	0.1510 (2)	−0.03792 (16)	0.29011 (13)	0.0230 (4)	
H6	0.0831	−0.1189	0.2730	0.028*	
C7	0.1206 (2)	0.05483 (15)	0.24141 (13)	0.0188 (4)	
H7	0.0334	0.0363	0.1915	0.023*	
C8	0.23227 (18)	0.69426 (14)	0.08443 (11)	0.0121 (3)	
C9	0.29777 (18)	0.83055 (14)	0.08443 (12)	0.0132 (3)	
C10	0.43016 (19)	0.89953 (15)	0.15359 (12)	0.0154 (3)	
H10	0.4770	0.8608	0.1992	0.018*	
C11	0.4919 (2)	1.02655 (15)	0.15411 (12)	0.0177 (3)	
C12	0.4219 (2)	1.08404 (15)	0.08613 (13)	0.0200 (4)	
H12	0.4629	1.1691	0.0867	0.024*	
C13	0.2912 (2)	1.01491 (15)	0.01772 (13)	0.0195 (4)	
H13	0.2447	1.0539	−0.0278	0.023*	
C14	0.22815 (19)	0.88799 (15)	0.01573 (12)	0.0163 (3)	
H14	0.1404	0.8420	−0.0309	0.020*	
C15	0.33791 (18)	0.49239 (15)	−0.07239 (12)	0.0140 (3)	
H15	0.3742	0.5730	−0.0413	0.017*	
C16	0.38521 (18)	0.46906 (15)	−0.16129 (12)	0.0139 (3)	
H16	0.4532	0.5324	−0.1887	0.017*	
C17	0.32938 (18)	0.34920 (15)	−0.20895 (12)	0.0129 (3)	
C18	0.23250 (19)	0.25737 (15)	−0.16302 (12)	0.0155 (3)	
H18	0.1951	0.1759	−0.1922	0.019*	
C19	0.19232 (19)	0.28830 (15)	−0.07373 (12)	0.0162 (3)	
H19	0.1279	0.2261	−0.0434	0.019*	
C20	0.36275 (19)	0.31412 (15)	−0.30949 (12)	0.0142 (3)	
C21	−0.13929 (18)	0.36943 (15)	0.24411 (12)	0.0146 (3)	
H21	−0.1718	0.2970	0.2005	0.018*	
C22	−0.21039 (19)	0.37093 (15)	0.32724 (12)	0.0149 (3)	
H22	−0.2885	0.3005	0.3390	0.018*	
C23	−0.16404 (18)	0.47843 (14)	0.39282 (11)	0.0120 (3)	
C24	−0.04688 (19)	0.58130 (15)	0.37131 (12)	0.0143 (3)	
H24	−0.0134	0.6553	0.4130	0.017*	
C25	0.01886 (19)	0.57159 (15)	0.28711 (12)	0.0144 (3)	
H25	0.0978	0.6404	0.2737	0.017*	
C26	−0.23947 (17)	0.47723 (14)	0.48447 (11)	0.0122 (3)	

### Atomic displacement parameters (Å^2^)

**Table:**

	*U*^11^	*U*^22^	*U*^33^	*U*^12^	*U*^13^	*U*^23^
Cd1	0.01097 (7)	0.00929 (7)	0.00898 (7)	0.00322 (5)	0.00236 (4)	0.00160 (4)
O1	0.0165 (6)	0.0152 (6)	0.0126 (6)	0.0056 (5)	−0.0011 (5)	0.0028 (4)
O2	0.0152 (6)	0.0119 (5)	0.0155 (6)	0.0033 (4)	0.0013 (5)	0.0026 (4)
O3	0.0189 (6)	0.0138 (6)	0.0182 (6)	0.0048 (5)	−0.0057 (5)	0.0024 (5)
O4	0.0140 (6)	0.0125 (5)	0.0166 (6)	0.0023 (4)	−0.0011 (5)	−0.0001 (4)
O5	0.0167 (6)	0.0126 (5)	0.0152 (6)	0.0036 (5)	−0.0007 (5)	0.0034 (4)
O6	0.0250 (7)	0.0150 (6)	0.0216 (7)	−0.0035 (5)	−0.0053 (5)	0.0003 (5)
O7	0.0201 (6)	0.0170 (6)	0.0177 (6)	0.0025 (5)	0.0033 (5)	−0.0030 (5)
O8	0.0175 (6)	0.0149 (6)	0.0151 (6)	0.0017 (5)	0.0069 (5)	0.0005 (4)
O9	0.0226 (7)	0.0173 (7)	0.0280 (7)	0.0016 (5)	−0.0025 (6)	0.0006 (5)
O10	0.0239 (7)	0.0191 (6)	0.0239 (7)	0.0078 (6)	0.0005 (5)	−0.0005 (5)
N1	0.0130 (6)	0.0154 (7)	0.0139 (7)	0.0059 (5)	0.0030 (5)	0.0027 (5)
N2	0.0180 (7)	0.0228 (8)	0.0112 (7)	0.0000 (6)	0.0052 (6)	−0.0042 (6)
N3	0.0127 (6)	0.0135 (7)	0.0114 (6)	0.0052 (5)	0.0021 (5)	0.0013 (5)
N4	0.0174 (7)	0.0157 (7)	0.0126 (7)	0.0028 (6)	0.0068 (6)	−0.0003 (5)
C1	0.0119 (7)	0.0147 (8)	0.0112 (7)	0.0055 (6)	0.0055 (6)	0.0026 (6)
C2	0.0137 (8)	0.0143 (8)	0.0120 (8)	0.0052 (6)	0.0039 (6)	0.0030 (6)
C3	0.0133 (8)	0.0122 (8)	0.0129 (8)	0.0032 (6)	0.0026 (6)	0.0010 (6)
C4	0.0155 (8)	0.0172 (8)	0.0119 (8)	0.0068 (7)	0.0006 (6)	0.0023 (6)
C5	0.0263 (9)	0.0142 (8)	0.0199 (9)	0.0064 (7)	−0.0009 (7)	0.0059 (7)
C6	0.0244 (9)	0.0120 (8)	0.0259 (10)	0.0005 (7)	−0.0045 (8)	0.0039 (7)
C7	0.0169 (8)	0.0173 (8)	0.0178 (8)	0.0028 (7)	−0.0042 (7)	0.0022 (7)
C8	0.0117 (7)	0.0133 (8)	0.0112 (7)	0.0030 (6)	0.0049 (6)	−0.0002 (6)
C9	0.0137 (8)	0.0128 (8)	0.0125 (8)	0.0034 (6)	0.0040 (6)	0.0007 (6)
C10	0.0172 (8)	0.0152 (8)	0.0130 (8)	0.0046 (7)	0.0021 (6)	0.0028 (6)
C11	0.0184 (8)	0.0148 (8)	0.0156 (8)	0.0005 (7)	0.0032 (7)	−0.0013 (6)
C12	0.0260 (9)	0.0098 (8)	0.0221 (9)	0.0024 (7)	0.0061 (7)	0.0025 (7)
C13	0.0248 (9)	0.0158 (8)	0.0191 (9)	0.0080 (7)	0.0030 (7)	0.0053 (7)
C14	0.0158 (8)	0.0154 (8)	0.0159 (8)	0.0039 (7)	0.0012 (6)	0.0004 (6)
C15	0.0126 (8)	0.0129 (8)	0.0158 (8)	0.0042 (6)	0.0011 (6)	0.0003 (6)
C16	0.0113 (7)	0.0148 (8)	0.0148 (8)	0.0027 (6)	0.0034 (6)	0.0035 (6)
C17	0.0109 (7)	0.0158 (8)	0.0131 (8)	0.0064 (6)	0.0011 (6)	0.0020 (6)
C18	0.0164 (8)	0.0121 (8)	0.0181 (8)	0.0051 (6)	0.0031 (7)	0.0011 (6)
C19	0.0168 (8)	0.0148 (8)	0.0185 (8)	0.0053 (6)	0.0065 (7)	0.0057 (7)
C20	0.0138 (8)	0.0168 (8)	0.0133 (8)	0.0075 (6)	0.0011 (6)	0.0018 (6)
C21	0.0138 (8)	0.0130 (8)	0.0153 (8)	0.0030 (6)	0.0023 (6)	−0.0015 (6)
C22	0.0135 (8)	0.0132 (8)	0.0160 (8)	0.0015 (6)	0.0036 (6)	0.0017 (6)
C23	0.0112 (7)	0.0139 (8)	0.0117 (7)	0.0057 (6)	0.0012 (6)	0.0014 (6)
C24	0.0149 (8)	0.0128 (8)	0.0143 (8)	0.0041 (6)	0.0022 (6)	−0.0003 (6)
C25	0.0149 (8)	0.0117 (8)	0.0163 (8)	0.0037 (6)	0.0037 (6)	0.0025 (6)
C26	0.0103 (7)	0.0157 (8)	0.0113 (7)	0.0059 (6)	0.0001 (6)	0.0018 (6)

### Geometric parameters (Å, °)

**Table:**

Cd1—O1	2.3029 (11)	C3—H3	0.9300
Cd1—O2	2.5562 (11)	C4—C5	1.387 (2)
Cd1—O4	2.4687 (11)	C5—C6	1.383 (2)
Cd1—O4^i^	2.3744 (11)	C5—H5	0.9300
Cd1—O5	2.3141 (11)	C6—C7	1.385 (2)
Cd1—N1	2.3256 (13)	C6—H6	0.9300
Cd1—N3	2.3238 (13)	C7—H7	0.9300
Cd1—C8	2.7525 (16)	C8—C9	1.492 (2)
O1—C1	1.2632 (19)	C9—C10	1.393 (2)
O2—C1	1.2624 (19)	C9—C14	1.390 (2)
O3—C4	1.374 (2)	C10—C11	1.390 (2)
O3—H3O	0.8200	C10—H10	0.9300
O4—Cd1^i^	2.3744 (11)	C11—C12	1.386 (3)
O4—C8	1.273 (2)	C12—C13	1.379 (2)
O5—C8	1.260 (2)	C12—H12	0.9300
O6—C11	1.362 (2)	C13—C14	1.387 (2)
O6—H6O	0.8200	C13—H13	0.9300
O7—C20	1.234 (2)	C14—H14	0.9300
O8—C26	1.2411 (19)	C15—C16	1.382 (2)
O9—H91	0.793 (16)	C15—H15	0.9300
O9—H92	0.783 (16)	C16—C17	1.389 (2)
O10—H101	0.806 (16)	C16—H16	0.9300
O10—H102	0.813 (16)	C17—C18	1.390 (2)
N1—C15	1.340 (2)	C17—C20	1.505 (2)
N1—C19	1.341 (2)	C18—C19	1.378 (2)
N2—C20	1.319 (2)	C18—H18	0.9300
N2—H2A	0.8600	C19—H19	0.9300
N2—H2B	0.8600	C21—C22	1.385 (2)
N3—C21	1.341 (2)	C21—H21	0.9300
N3—C25	1.338 (2)	C22—C23	1.388 (2)
N4—C26	1.323 (2)	C22—H22	0.9300
N4—H4A	0.8600	C23—C24	1.394 (2)
N4—H4B	0.8600	C23—C26	1.508 (2)
C1—C2	1.495 (2)	C24—C25	1.382 (2)
C2—C3	1.391 (2)	C24—H24	0.9300
C2—C7	1.387 (2)	C25—H25	0.9300
C3—C4	1.384 (2)		
			
O1—Cd1—O2	53.71 (4)	C5—C6—H6	119.6
O1—Cd1—O4	165.84 (4)	C7—C6—H6	119.6
O1—Cd1—O4^i^	91.05 (4)	C2—C7—H7	120.3
O1—Cd1—O5	138.24 (4)	C6—C7—C2	119.42 (16)
O1—Cd1—N1	86.48 (4)	C6—C7—H7	120.3
O1—Cd1—N3	89.70 (4)	O4—C8—Cd1	63.72 (8)
O1—Cd1—C8	164.83 (4)	O4—C8—C9	120.21 (14)
O2—Cd1—C8	112.50 (4)	O5—C8—O4	120.37 (14)
O4—Cd1—O2	139.97 (4)	O5—C8—Cd1	56.69 (8)
O4^i^—Cd1—O2	143.60 (4)	O5—C8—C9	119.41 (14)
O4^i^—Cd1—O4	75.96 (4)	C9—C8—Cd1	175.33 (11)
O4—Cd1—C8	27.53 (4)	C10—C9—C8	119.28 (14)
O4^i^—Cd1—C8	103.46 (4)	C14—C9—C8	120.38 (14)
O5—Cd1—O2	85.54 (4)	C14—C9—C10	120.34 (15)
O5—Cd1—O4	54.59 (4)	C9—C10—H10	120.2
O5—Cd1—O4^i^	130.53 (4)	C11—C10—C9	119.65 (15)
O5—Cd1—N1	94.01 (4)	C11—C10—H10	120.2
O5—Cd1—N3	92.07 (4)	O6—C11—C10	122.77 (16)
O5—Cd1—C8	27.07 (4)	O6—C11—C12	117.16 (15)
N1—Cd1—O2	101.27 (4)	C12—C11—C10	120.05 (15)
N1—Cd1—O4	86.39 (4)	C11—C12—H12	120.1
N1—Cd1—O4^i^	83.17 (4)	C13—C12—C11	119.87 (15)
N1—Cd1—C8	90.85 (4)	C13—C12—H12	120.1
N3—Cd1—O2	80.25 (4)	C12—C13—C14	120.96 (16)
N3—Cd1—O4	96.29 (4)	C12—C13—H13	119.5
N3—Cd1—O4^i^	92.07 (4)	C14—C13—H13	119.5
N3—Cd1—N1	173.83 (4)	C9—C14—H14	120.4
N3—Cd1—C8	94.08 (4)	C13—C14—C9	119.12 (15)
C1—O1—Cd1	98.12 (9)	C13—C14—H14	120.4
C1—O2—Cd1	86.39 (9)	N1—C15—C16	123.15 (15)
C4—O3—H3O	109.5	N1—C15—H15	118.4
C8—O4—Cd1^i^	166.94 (11)	C16—C15—H15	118.4
C8—O4—Cd1	88.75 (9)	C15—C16—C17	118.76 (14)
Cd1^i^—O4—Cd1	104.04 (4)	C15—C16—H16	120.6
C8—O5—Cd1	96.24 (9)	C17—C16—H16	120.6
C11—O6—H6O	109.5	C16—C17—C18	118.20 (14)
H91—O9—H92	115 (2)	C16—C17—C20	123.58 (14)
H101—O10—H102	108 (2)	C18—C17—C20	118.18 (14)
C15—N1—Cd1	123.35 (11)	C17—C18—H18	120.3
C15—N1—C19	117.84 (14)	C19—C18—C17	119.34 (15)
C19—N1—Cd1	117.64 (10)	C19—C18—H18	120.3
C20—N2—H2A	120.0	N1—C19—C18	122.68 (15)
C20—N2—H2B	120.0	N1—C19—H19	118.7
H2A—N2—H2B	120.0	C18—C19—H19	118.7
C25—N3—Cd1	121.36 (10)	O7—C20—N2	123.91 (15)
C25—N3—C21	117.79 (13)	O7—C20—C17	119.22 (14)
C21—N3—Cd1	119.83 (10)	N2—C20—C17	116.84 (14)
C26—N4—H4A	120.0	N3—C21—C22	122.72 (15)
C26—N4—H4B	120.0	N3—C21—H21	118.6
H4A—N4—H4B	120.0	C22—C21—H21	118.6
O1—C1—C2	118.58 (14)	C21—C22—C23	119.42 (14)
O2—C1—O1	121.78 (14)	C21—C22—H22	120.3
O2—C1—C2	119.64 (14)	C23—C22—H22	120.3
C3—C2—C1	119.07 (14)	C22—C23—C24	117.88 (14)
C7—C2—C1	120.68 (14)	C22—C23—C26	118.64 (14)
C7—C2—C3	120.24 (15)	C24—C23—C26	123.46 (14)
C2—C3—H3	120.1	C23—C24—H24	120.5
C4—C3—C2	119.70 (15)	C25—C24—C23	118.98 (14)
C4—C3—H3	120.1	C25—C24—H24	120.5
O3—C4—C3	121.96 (14)	N3—C25—C24	123.21 (15)
O3—C4—C5	117.70 (14)	N3—C25—H25	118.4
C3—C4—C5	120.34 (15)	C24—C25—H25	118.4
C4—C5—H5	120.2	O8—C26—N4	122.24 (14)
C6—C5—C4	119.53 (16)	O8—C26—C23	119.56 (14)
C6—C5—H5	120.2	N4—C26—C23	118.19 (14)
C5—C6—C7	120.76 (16)		
			
O2—Cd1—O1—C1	0.07 (8)	N3—Cd1—C8—O5	86.55 (9)
O4—Cd1—O1—C1	166.71 (14)	Cd1—O1—C1—O2	−0.14 (16)
O4^i^—Cd1—O1—C1	−170.08 (9)	Cd1—O1—C1—C2	179.96 (11)
O5—Cd1—O1—C1	14.75 (12)	Cd1—O2—C1—O1	0.13 (14)
N1—Cd1—O1—C1	106.82 (9)	Cd1—O2—C1—C2	−179.97 (13)
N3—Cd1—O1—C1	−78.02 (9)	Cd1^i^—O4—C8—Cd1	168.5 (5)
C8—Cd1—O1—C1	26.6 (2)	Cd1—O4—C8—O5	2.25 (14)
O1—Cd1—O2—C1	−0.07 (8)	Cd1^i^—O4—C8—O5	170.7 (4)
O4—Cd1—O2—C1	−175.03 (8)	Cd1—O4—C8—C9	−177.15 (13)
O4^i^—Cd1—O2—C1	16.67 (12)	Cd1^i^—O4—C8—C9	−8.7 (5)
O5—Cd1—O2—C1	−170.33 (9)	Cd1—O5—C8—O4	−2.41 (15)
N1—Cd1—O2—C1	−77.12 (9)	Cd1—O5—C8—C9	176.99 (12)
N3—Cd1—O2—C1	96.80 (9)	Cd1—N1—C15—C16	−166.50 (12)
C8—Cd1—O2—C1	−172.80 (8)	C19—N1—C15—C16	0.8 (2)
O1—Cd1—O4—Cd1^i^	23.96 (17)	Cd1—N1—C19—C18	166.50 (13)
O1—Cd1—O4—C8	−158.70 (14)	C15—N1—C19—C18	−1.5 (2)
O2—Cd1—O4—Cd1^i^	−172.88 (4)	Cd1—N3—C21—C22	168.08 (12)
O2—Cd1—O4—C8	4.46 (11)	C25—N3—C21—C22	−0.5 (2)
O4^i^—Cd1—O4—Cd1^i^	0.0	Cd1—N3—C25—C24	−168.47 (12)
O4^i^—Cd1—O4—C8	177.33 (11)	C21—N3—C25—C24	0.0 (2)
O5—Cd1—O4—Cd1^i^	−178.63 (6)	O1—C1—C2—C3	169.40 (14)
O5—Cd1—O4—C8	−1.29 (8)	O1—C1—C2—C7	−9.2 (2)
N1—Cd1—O4—Cd1^i^	83.86 (5)	O2—C1—C2—C3	−10.5 (2)
N1—Cd1—O4—C8	−98.81 (9)	O2—C1—C2—C7	170.87 (15)
N3—Cd1—O4—Cd1^i^	−90.57 (5)	C1—C2—C3—C4	−177.44 (14)
N3—Cd1—O4—C8	86.77 (9)	C7—C2—C3—C4	1.2 (2)
C8—Cd1—O4—Cd1^i^	−177.33 (11)	C1—C2—C7—C6	178.17 (15)
O1—Cd1—O5—C8	173.20 (8)	C3—C2—C7—C6	−0.4 (3)
O2—Cd1—O5—C8	−174.98 (9)	C2—C3—C4—O3	178.19 (14)
O4—Cd1—O5—C8	1.31 (8)	C2—C3—C4—C5	−1.0 (2)
O4^i^—Cd1—O5—C8	−0.43 (11)	O3—C4—C5—C6	−179.20 (16)
N1—Cd1—O5—C8	84.00 (9)	C3—C4—C5—C6	0.0 (3)
N3—Cd1—O5—C8	−94.93 (9)	C4—C5—C6—C7	0.8 (3)
O1—Cd1—N1—C15	−159.66 (12)	C5—C6—C7—C2	−0.6 (3)
O1—Cd1—N1—C19	33.01 (12)	O4—C8—C9—C10	179.21 (14)
O2—Cd1—N1—C15	−107.75 (12)	O4—C8—C9—C14	−1.2 (2)
O2—Cd1—N1—C19	84.92 (12)	O5—C8—C9—C10	−0.2 (2)
O4—Cd1—N1—C15	32.58 (12)	O5—C8—C9—C14	179.36 (14)
O4^i^—Cd1—N1—C15	108.86 (12)	C8—C9—C10—C11	180.00 (14)
O4—Cd1—N1—C19	−134.74 (12)	C14—C9—C10—C11	0.4 (2)
O4^i^—Cd1—N1—C19	−58.47 (12)	C8—C9—C14—C13	179.76 (14)
O5—Cd1—N1—C15	−21.51 (12)	C10—C9—C14—C13	−0.7 (2)
O5—Cd1—N1—C19	171.16 (12)	C9—C10—C11—O6	−178.74 (15)
C8—Cd1—N1—C15	5.40 (12)	C9—C10—C11—C12	0.1 (2)
C8—Cd1—N1—C19	−161.93 (12)	O6—C11—C12—C13	178.56 (16)
O1—Cd1—N3—C21	−31.57 (12)	C10—C11—C12—C13	−0.3 (3)
O1—Cd1—N3—C25	136.62 (12)	C11—C12—C13—C14	0.0 (3)
O2—Cd1—N3—C21	−84.73 (12)	C12—C13—C14—C9	0.4 (3)
O2—Cd1—N3—C25	83.47 (12)	N1—C15—C16—C17	1.1 (2)
O4—Cd1—N3—C21	135.57 (12)	C15—C16—C17—C18	−2.2 (2)
O4^i^—Cd1—N3—C21	59.47 (12)	C15—C16—C17—C20	175.29 (15)
O4—Cd1—N3—C25	−56.24 (12)	C16—C17—C18—C19	1.5 (2)
O4^i^—Cd1—N3—C25	−132.34 (12)	C20—C17—C18—C19	−176.10 (15)
O5—Cd1—N3—C21	−169.84 (12)	C16—C17—C20—O7	−157.95 (16)
O5—Cd1—N3—C25	−1.64 (12)	C16—C17—C20—N2	20.1 (2)
C8—Cd1—N3—C21	163.13 (12)	C18—C17—C20—O7	19.5 (2)
C8—Cd1—N3—C25	−28.68 (12)	C18—C17—C20—N2	−162.40 (15)
O1—Cd1—C8—O4	160.15 (13)	C17—C18—C19—N1	0.4 (3)
O1—Cd1—C8—O5	−17.5 (2)	N3—C21—C22—C23	0.4 (3)
O2—Cd1—C8—O4	−176.90 (8)	C21—C22—C23—C24	0.2 (2)
O2—Cd1—C8—O5	5.42 (10)	C21—C22—C23—C26	−178.24 (14)
O4^i^—Cd1—C8—O4	−2.66 (11)	C22—C23—C24—C25	−0.8 (2)
O4—Cd1—C8—O5	−177.68 (15)	C26—C23—C24—C25	177.64 (15)
O4^i^—Cd1—C8—O5	179.66 (9)	C22—C23—C26—O8	4.0 (2)
O5—Cd1—C8—O4	177.68 (15)	C22—C23—C26—N4	−177.01 (15)
N1—Cd1—C8—O4	80.52 (9)	C24—C23—C26—O8	−174.35 (15)
N1—Cd1—C8—O5	−97.16 (9)	C24—C23—C26—N4	4.6 (2)
N3—Cd1—C8—O4	−95.77 (9)	C23—C24—C25—N3	0.7 (2)

Symmetry codes: (i) −*x*, −*y*+1, −*z*.

### Hydrogen-bond geometry (Å, °)

**Table:**

*D*—H···*A*	*D*—H	H···*A*	*D*···*A*	*D*—H···*A*
N2—H2A···O8^ii^	0.86	2.12	2.9158 (19)	153
N2—H2B···O2^iii^	0.86	2.00	2.8290 (18)	160
O3—H3O···O8^iv^	0.82	1.91	2.7248 (16)	173
N4—H4A···O2^v^	0.86	2.20	3.0404 (18)	164
N4—H4B···O9^vi^	0.86	2.01	2.846 (2)	166
O6—H6O···O10^vi^	0.82	1.91	2.7043 (18)	163
O9—H91···O10^vii^	0.79 (2)	2.03 (2)	2.819 (2)	173 (2)
O9—H92···O7^viii^	0.78 (2)	2.29 (2)	2.9508 (19)	143 (2)
O10—H101···O3^vii^	0.81 (2)	2.17 (2)	2.8976 (19)	150 (2)
O10—H102···O7^ix^	0.81 (2)	1.89 (2)	2.6876 (18)	165 (3)
C14—H14···O1^i^	0.93	2.28	3.197 (2)	171
C19—H19···O1	0.93	2.54	3.161 (2)	124
C21—H21···O6^x^	0.93	2.49	3.128 (2)	126
C22—H22···O6^x^	0.93	2.58	3.163 (2)	121
C24—H24···O9^vi^	0.93	2.32	3.222 (2)	164

Symmetry codes: (ii) *x*+1, *y*, *z*−1; (iii) −*x*+1, −*y*+1, −*z*; (iv) *x*+1, *y*, *z*; (v) −*x*, −*y*+1, −*z*+1; (vi) −*x*+1, −*y*+1, −*z*+1; (vii) −*x*+1, −*y*, −*z*+1; (viii) *x*+1, *y*, *z*+1; (ix) *x*, *y*, *z*+1; (i) −*x*, −*y*+1, −*z*; (x) *x*−1, *y*−1, *z*.
